# Temporo-Spectral Imaging of Intrinsic Optical Signals during Hypoxia-Induced Spreading Depression-Like Depolarization

**DOI:** 10.1371/journal.pone.0043981

**Published:** 2012-08-29

**Authors:** Maria Mané, Michael Müller

**Affiliations:** DFG Research Center Molecular Physiology of the Brain (CMPB), Zentrum für Physiologie und Pathophysiologie, Abteilung Neuro- und Sinnesphysiologie, Georg-August-Universität Göttingen, Göttingen, Germany; University G. D’Annunzio, Italy

## Abstract

Spreading depression (SD) is characterized by a sustained near-complete depolarization of neurons, a massive depolarization of glia, and a negative deflection of the extracellular DC potential. These electrophysiological signs are accompanied by an intrinsic optical signal (IOS) which arises from changes in light scattering and absorption. Even though the underlying mechanisms are unclear, the IOS serves as non-invasive tool to define the spatiotemporal dynamics of SD in brain slices. Usually the tissue is illuminated by white light, and light reflectance or transmittance is monitored. Using a polychromatic, fast-switchable light source we now performed temporo-spectral recordings of the IOS associated with hypoxia-induced SD-like depolarization (HSD) in rat hippocampal slices kept in an interface recording chamber. Recording full illumination spectra (320–680 nm) yielded distinct reflectance profiles for the different phases of HSD. Early during hypoxia tissue reflectance decreased within almost the entire spectrum due to cell swelling. HSD was accompanied by a reversible reflectance increase being most pronounced at 400 nm and 460 nm. At 440 nm massive porphyrin absorption (Soret band) was detected. Hypotonic solutions, Ca^2+^-withdrawal and glial poisoning intensified the reflectance increase during HSD, whereas hypertonic solutions dampened it. Replacement of Cl^-^ inverted the reflectance increase. Inducing HSD by cyanide distorted the IOS and reflectance at 340–400 nm increased irreversibly. The pronounced changes at short wavelengths (380 nm, 460 nm) and their cyanide sensitivity suggest that block of mitochondrial metabolism contributes to the IOS during HSD. For stable and reliable IOS recordings during HSD wavelengths of 460–560 nm are recommended.

## Introduction

Spreading depression (SD) is a depolarizing wave that slowly propagates within the gray matter and temporarily shuts down neuronal function and information processing within neuronal networks (for review see [1], [2], [3], [4]). There is convincing evidence that the occurrence of this phenomenon is linked to certain conditions of disturbed brain function as well as brain pathology such as brain injury, edema, hemorrhage, epilepsy, migraine and stroke [5], [6], [7], [8], [9], [10]. Even though the massive electrophysiological, ionic, metabolic and hemodynamic changes associated with SD are fully reversible, its repetitive occurrence and especially long lasting SD episodes are thought to harm brain tissue and to worsen the outcome of brain pathology [4], [8], [11], [12], [13], [14]. Accordingly there is an enormous interest in both the mechanistic analysis of SD and the reliable monitoring of its occurrence in animal models and patients.

Such monitoring can be performed by either electrical recordings or by taking advantage of the optical signature of SD, the so-called intrinsic optical signal (IOS) [15], [16], [17], [18], [19], [20]. Each approach has its specific advantages and disadvantages. Electrical recordings yield superior temporal resolution, but to obtain spatial information electrodes have to be inserted at distinct sites which may cause microdamage. Optical monitoring usually provides a lower temporal resolution, but it is non-invasive and offers the advantage of overlooking larger brain areas and yielding reliable spatiotemporal information on SD propagation. Hence there is an increasing use of the optical approach, often in combination with electrophysiological recordings from defined locations to benefit from the mutual complementation of the specific advantages of both techniques. To list just a few examples, we have used IOS analyses in the past in isolated rodent brain tissue to confirm that SD also occurs in brainstem [21], to analyze its propagation pattern in neurodevelopmental disorders [22], [23], and to define the impact of drugs on SD onset and propagation [24], [25], [26]. An IOS with very similar properties to that in rodent preparations has also been recorded in human brain slices [27], [28]). Others have even performed optical recordings of light scattering and/or NADH autofluorescence in patients during surgical interventions [29] or to verify the occurrence of SD after severe head injury [30]. Subdural opto-electrode strips are already in use to define the detailed consequences of SD in brain pathology [2], and in the future they may open the opportunity to monitor in addition to electrophysiological responses also the IOS from patients on a regular basis.

The IOS is composed of a variety of optical alterations that can be measured within neural tissue without the need of adding optical indicators such as fluorescent dyes or proteins. Its detailed components depend on the type of preparation used, the very experimental conditions, and especially the illumination wavelengths chosen. Under *in vivo* conditions the IOS is dominated by changes in blood flow and hemoglobin oxygenation [31], [32], whereas changes in light scattering dominate in isolated preparations [15], [16], [17], [18], [19], [20]. Thus it is not surprising that in the past differing or even seemingly opposite observations have been obtained in identical or very similar preparations.

Despite being used widely and successfully, the detailed mechanisms underlying the generation of the IOS during SD are still only partly understood. In the early days of IOS imaging cell volume changes had been proposed to be a major component [18], [19], yet this theory was later challenged by the observations that the replacement of Cl^−^ largely depressed the IOS but did not prevent cell swelling [16] and that scattering changes evoked by osmolarity changes are of opposite sign as those associated with SD [20]. Due to the irreversible nature of some IOS components also irreversible neuronal damage such as dendritic beading was proposed to contribute to the optical changes [17].

With changes in light scattering underlying the gross amount of the IOS it is reasonable to assume that also changes in cellular components and organelles are involved [33]. Accordingly, earlier studies suggested that also mitochondria may contribute to the generation of the IOS. Due to the close correlation between mitochondrial depolarization and the IOS associated with SD and HSD [34], this could involve changes in mitochondrial microarchitecture and/or metabolic activity [16], [26], [34]. Furthermore, metabolic alterations could give rise to a modified light absorption by the porphyrin-containing cytochromes within the mitochondrial respiratory chain [35], [36], [37].

It is important to note that the IOS during SD clearly differs from the IOS associated with synaptic or neuronal activity. The activity-related IOS is of opposite sign and an order of magnitude less intense. It is Cl^−^dependent, sensitive to Ca^2+^-withdrawal, and there is general agreement that it is arises from cell volume changes, i.e., cell swelling [38], [39].

To improve the understanding of the IOS associated with SD in more detail, we have now performed multispectral IOS recordings using a rapidly-switchable polychromatic light source for the defined multispectral illumination of the tissue. In acute rat hippocampal slices, the changes in light reflectance associated with hypoxia-induced SD-like depolarization (HSD) were monitored continuously while illuminating the tissue with a total of 19 defined wavelengths in the range of 320–680 nm. These temporo-spectral recordings provide a complex optical signature of HSD that showed characteristic alterations in response to changes in osmolarity of the solutions or their ionic composition as well as the pharmacological targeting of mitochondria. These spectrally-defined optical fingerprints of HSD yield new insights into the mechanisms underlying the generation of the IOS in isolated brain tissue. At the same time they define conditions for the stable recording of reliable IOS responses associated with SD under various experimental conditions.

## Materials and Methods

### Preparation

All experiments were performed in accordance with local regulations and the ethical guidelines for the care and use of laboratory animals (U.S. National Institutes of Health). The entire study was performed on isolated brain tissue. Animal dissection procedures and anesthesia were approved by the Office of Animal Welfare of the University of Göttingen (file number T-13.08). Hippocampal tissue slices were prepared from ether anesthetized male Sprague-Dawley rats of 180–300g body weight (5–10 weeks old) as described in detail earlier [40]. In brief, following decapitation the brain was rapidly removed from the skull and placed in ice-cold artificial cerebrospinal fluid (ACSF, for composition see below) for a few minutes. The hemispheres were separated, and 400 µm-thick tissue slices were cut using a vibroslicer (Campden Instruments, 752M Vibroslice). Slices were then transferred to an interface recording chamber (Oslo style) and left undisturbed for at least 90 min. The recording chamber was kept at a temperature of 35–36°C, aerated with 95% O_2_ - 5% CO_2_ (carbogen, 400 ml/min), and perfused with either oxygenated ACSF or the respective experimental solutions (3–4 ml/min). Severe hypoxia was induced by switching the chamber’s gas supply to 95% N_2_ - 5% CO_2_; during that time, oxygenation of the experimental solutions was continued.

### Solutions

All chemicals - unless otherwise mentioned - were obtained from Sigma-Aldrich. The ACSF was composed of (in mM): 130 NaCl, 3.5 KCl, 1.25 NaH_2_PO_4_, 24 NaHCO_3_, 1.2 CaCl_2_, 1.2 MgSO_4_, and 10 dextrose; it was constantly aerated with carbogen to adjust the pH to 7.4. In the case of nominally-free Ca^2+^ solution, CaCl_2_ was omitted. In the low-Cl^−^ solution 130 mM NaCl were replaced by the membrane-impermeable anion methylsulfate [41], [42] that was obtained as sodium salt (TCI Europe). CN^−^ (cyanide, sodium salt) was dissolved as an aqueous 1 M stock, FCCP (carbonyl cyanide 4-(trifluoromethoxy) phenylhydrazone, Tocris) was dissolved in DMSO (dimethyl sulfoxide) as a 10 mM stock, and both solutions were kept frozen. FAc (fluoroacetate) was directly added to the ACSF.

### DC-potential Recordings

**Figure 1:**
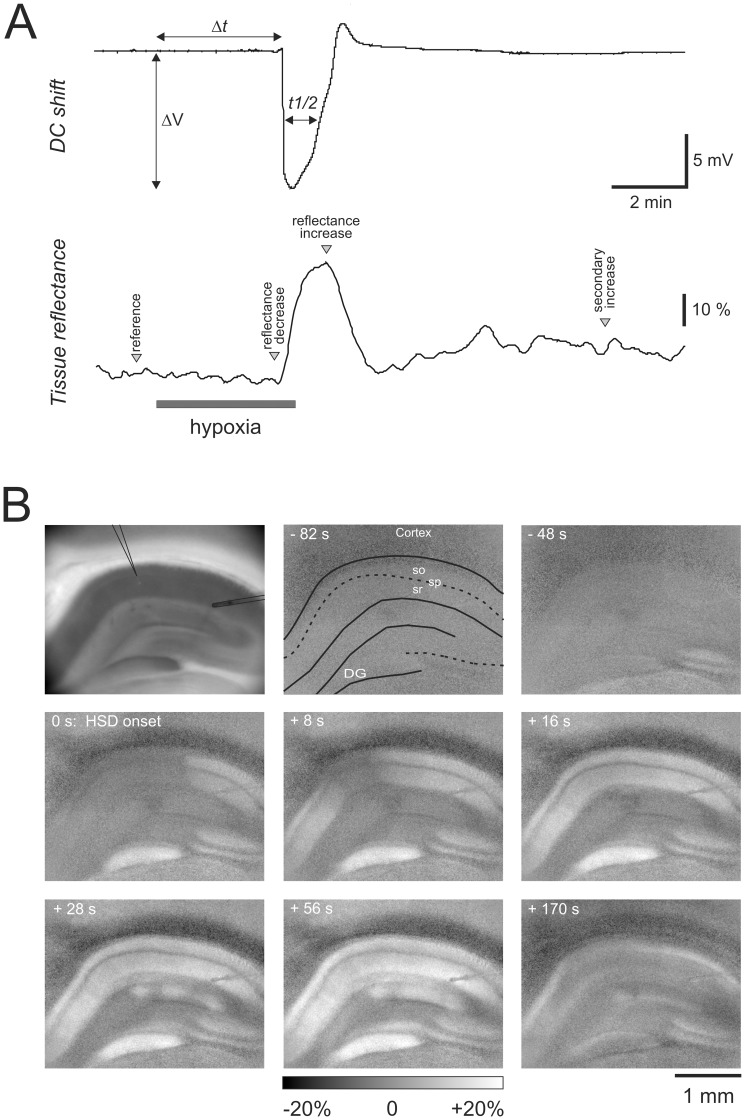
Electrical and optical signs of HSD in acute hippocampal tissue slices. A ) HSD-associated extracellular DC potential shift recorded in *stratum radiatum* of the CA1 subfield. The characteristic parameters of the DC potential deflection, amplitude (ΔV), time to onset (Δt), and duration at half maximum amplitude (t½) are defined. The lower trace shows the changes in light reflectance associated with HSD during illumination of the tissue with white light. Reflectance changes were quantified in a small region of interest within *stratum radiatum* nearby the recording electrode. Spectral analyses were performed at the indicated characteristic time points: minimum reflectance shortly before HSD onset, maximum reflectance during HSD, and reflectance after recovery from HSD, 8 min upon reoxygenation. **B**) Spatiotemporal pattern of the IOS associated with HSD. The first image shows the slice analyzed, and the positions of the recording electrode (center) and a stimulation electrode (right side) are indicated. The following images are subtraction images showing the changes in light reflectance within the tissue as referred to the normoxic baseline reflectance. Reflectance changes are displayed in 256 gray-scale levels (8 bit) spanning a full-scale range of ±20% brightness changes. Numbers indicate the time relative to the occurrence of HSD at the recording electrode (t = 0). Some anatomical landmarks are indicated (so, *stratum oriens*, sp *stratum pyramidale*, sr *stratum radiatum, DG dentate gyrus*).

Glass microelectrodes for extracellular recordings were pulled from thin-walled borosilicate glass (GC150TF-10, Harvard Apparatus) using a horizontal electrode-puller (P-97, Sutter Instruments). They were filled with ACSF and their tips were trimmed to a final resistance of 5–10 MΩ. DC potentials were recorded with a custom built amplifier in *stratum radiatum* of the CA1 subfield [25], [40], digitized by a Digitizer 1322A acquisition system at sampling rates of 100 Hz, and analyzed offline with PClamp 9.0 software (Molecular Devices, Foster City, CA). All signal amplitudes were measured between the normoxic baseline and the maximal change (see Fig. 1A for a definition of the analyzed parameters). Only rapid DC potential deflections of at least -10 mV amplitude were considered as HSD, and only those slices were accepted, which produced an HSD under control conditions no earlier than 90 s after O_2_ withdrawal. HSD onset was defined as the occurrence of the sudden DC potential deflection.

### Optical Recordings

The characteristic IOS, i.e., the changes in light scattering during hypoxia and HSD were monitored with a computer-controlled imaging system composed of a xenon high stability light source (Polychrome V, Till Photonics) and a CCD camera (Imago QE, PCO Imaging). Taking advantage of the rapid wavelength-switching capabilities of this system (up to 400 nm/ms), the illumination wavelength of the tissue was varied in 20 nm increments from 320 to 680 nm and reflection images of the slice were taken at each wavelength with an exposure time of 30 ms and 4x4 pixel binning. With an 100 ms delay implemented in between the single wavelength exposures, an illumination spectrum was acquired within ∼2.4 s. Slices were illuminated at an angle of 45° and illumination spectra were recorded every 15 s. Slices were viewed with a 5x, 0.13NA objective (Epiplan, Zeiss) and an Axiotech microscope (Zeiss). To observe the entire hippocampal formation, a 0.5X C-mount CCD camera adapter was used. Reflectance changes induced by hypoxia or HSD were visualized by offline image subtraction and referred (normalized) to the control spectrum taken before O_2_ withdrawal [16]. The temporo-spectral profile of the optical changes was quantified in a rectangular region of interest close to the recording electrode. Image processing was performed with TILLvision 4.5 (TILL Photonics) and Metamorph 7.0 (Universal Imaging Corporation).

### Statistics

The presented data were obtained from 77 rats, using up to 4 slices from each brain. To ensure independence of observations, each treatment was performed on at least 3 different rats. All numerical values are given as mean ± standard deviation. Drug- or treatment-induced changes were normalized to control conditions (ACSF), by calculating the differences (ratio) of the 2^nd^ and 1^st^ HSD recorded in every slice. Statistical significance of the electrophysiological and optical data was tested in one-sample Student´s t-tests at a significance level of 5%, by comparing these differences against pre-treatment control conditions defined as unity [43]. This approach circumvents the problem of multiple comparisons and allows the use of statistical tests with a high discriminatory power. In those slices which underwent FAc-pretreatment without a prior control HSD, the data recorded in the presence of this drug were directly compared to the control group, i.e., all 1^st^ HSD episodes recorded under control conditions, using a two-tailed unpaired student´s tests. In the diagrams significant changes are indicated by asterisks (* P<0.05; ** P<0.01). For data processing and statistical calculations Excel software (Office 2007), Sigma Plot 10.01 (Systat Software) and Sigma Stat 3.5 (Systat Software) were used.

## Results

So far, the IOS associated with SD and HSD has usually been recorded using white light for the illumination/transillumination of the tissue of interest. We have now performed multispectral recordings to obtain more detailed insights into the properties of the IOS and its underlying cellular/molecular mechanisms. Acute rat hippocampal tissue slices were illuminated with a rapid sequence of wavelengths ranging from 320 to 680 nm. To minimize tissue illumination, full reflectance spectra were recorded every 15 s only. In addition the extracellular DC potential was monitored to define the occurrence and time course of HSD. The recording electrode was placed in *stratum radiatum* of the CA1 subfield and the changes in tissue reflectance were quantified within a rectangular region of interest (∼50x50 µm) nearby the recording electrode. To account for differences in the individual slices and the spectral properties of the xenon light bulb, changes in tissue reflectance were referred (normalized) to the normoxic baseline reflectance of the respective slice.

### Properties of the Multispectral IOS

In hippocampal slices kept under control conditions, O_2_ withdrawal induced HSD within 2.7±0.9 min, as judged by the occurrence of the sudden, negative DC potential deflection. The amplitude of the DC potential shift averaged −15.8±3.9 mV and measured at its half maximum amplitude it lasted 59.4±23.3 s (n = 52). To ensure full recovery, O_2_ was resubmitted 30 s after the onset of the DC potential shift, which then slowly recovered to its prehypoxic baseline (Fig. 1A). As a reference, and to define the specific time points analyzed in detail, the changes in light reflectance during HSD shall first be described which are obtained, when slices are illuminated “in the usual way”, i.e., by white light. Under these conditions a characteristic profile of reflectance changes was observed, and – as defined for the first time in the isolated chick retina [44] – four different phases can be distinguished: HSD was preceded by a moderate and transient decrease in tissue reflectance, i.e., “darkening” of the slice (phase 1). As soon as the characteristic DC potential shift occurred, tissue reflectance started to increase by up to 30%, causing a “brightening” of the slice (phase 2) which reached its maximum typically within 30–40s upon HSD onset. It is especially this marked reflectance increase which visualizes the spatiotemporal profile of HSD propagation and identifies unequivocally those tissue areas being invaded (Fig. 1B). Upon reoxygenation, tissue reflectance recovered to almost prehypoxic baseline conditions (phase 3), and then a slowly developing and moderate secondary reflectance increase occurred (phase 4).

**Figure 2:**
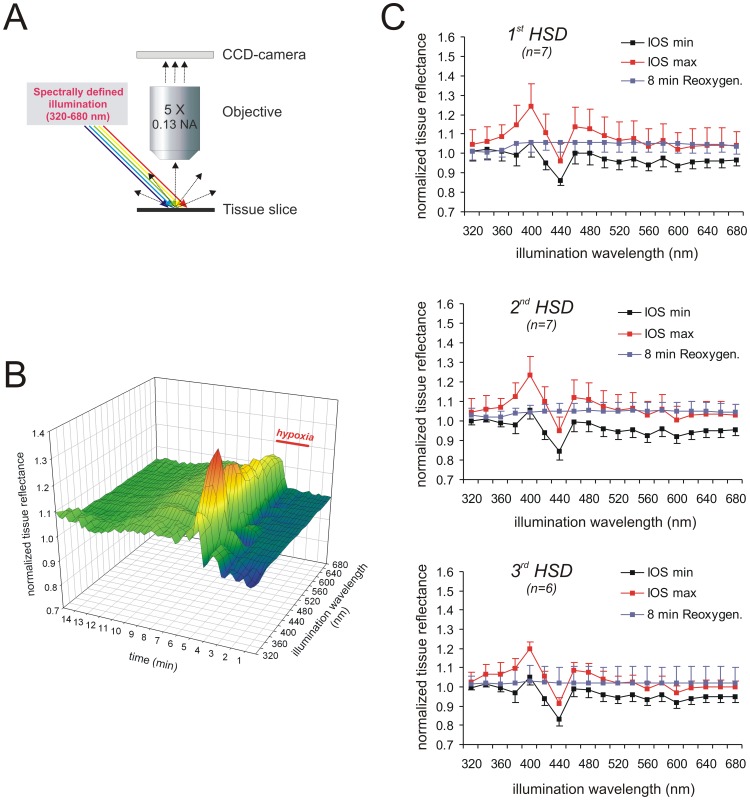
Temporo-spectral properties of the IOS. A ) For multispectral IOS recordings the slice was illuminated at an angle of 45°. The illumination wavelength was varied from 320 nm to 680 nm in 20-nm increments, and a full reflectance spectrum was recorded every 15 s. **B**) Sample recording of the multispectral IOS associated with HSD under control conditions. The changes in light reflectance are normalized to normoxic baseline reflectance and displayed as a surface plot by plotting them versus the respective wavelength and time. The duration of hypoxia is indicated. **C**) Averaged reflectance profiles obtained at the 3 defined time points (see Fig. 1A) during repeated hypoxia: minimum reflectance early during hypoxia before HSD onset (IOS min), maximum reflectance increase during HSD (IOS max), secondary reflectance increase during posthypoxic recovery 8 min after oxygenation was restored. Note that repeated hypoxia separated by 20 min of recovery did not affect the averaged reflectance profiles. The number of slices analyzed is reported; error bars represent standard deviations.

For the multispectral IOS recordings, spectrally-defined illumination with a half-power spectral bandwidth of 15 nm was used (Fig. 2A), and the obtained spectral signature of light reflectance was visualized as a surface plot. It shows the normalized reflectance changes at the 19 different wavelengths taken every 15 s during the course of the experiment and starts with 2 min of normoxic baseline recordings before O_2_ was withdrawn (Fig. 2B). In addition, the reflectance changes were averaged for the three characteristic time points/phases defined in Fig. 1A, and all further data presentations follow along this chronological time line (Fig. 2C):

- minimum reflectance during early hypoxia, before HSD onset (phase 1)
- maximum reflectance during HSD (phase 2)
- secondary reflectance increase, 8 min following reoxygenation (phase 4)

Early during hypoxia, before HSD onset, tissue reflectance slightly decreased over almost the entire wavelength range, with the most pronounced reflectance decrease (−14.3±4.3%, n = 52) occurring at 440 nm; only at 400 nm a moderate reflectance increase was observed consistently (Fig. 2B). The onset of HSD was paralleled by a very clear reflectance increase and two well pronounced peaks developed at 400 and 460 nm, at which reflectance rose by an average of 22.0±10.4% and 12.4±9.7%, respectively. At a wavelength of 440 nm a pronounced decrease in tissue reflectance occurred which even undershot the normoxic baseline by −2.9±8.9% (n = 52). On average, the most intense reflectance changes were detected 30–60s after the onset of the DC potential shift. Upon reoxygenation, the HSD-induced reflectance changes recovered within a few minutes, and a weak secondary increase in tissue reflectance then developed at wavelengths ≥380 nm (Fig. 2B).

To define the consequences of repeated hypoxia, 3 successive HSD episodes were induced, each separated by 20 min of posthypoxic recovery. Repeatedly inducing HSD is known to mediate only very moderate effects on the electrical signs of HSD [24]. Accordingly, the DC potential shift remained largely unaffected (summarized in Table 1) and only during the 3^rd^ HSD, its duration was slightly decreased. The multispectral IOS did not show any significant changes. The general profile of the surface plots and the reflectance profiles at the three defined time points (shortly before HSD, peak of HSD, posthypoxic recovery) remained largely unchanged (n = 6–7, Fig. 2C). With repeated hypoxia, the magnitude of the optical changes during HSD tended to decrease slightly, and the noticeable reflectance decrease at 440 nm tended to become more intense early during hypoxia and at the peak of HSD. Also the secondary reflectance increase during posthypoxic recovery tended to become less intense. The level of significance was, however, not reached for any of these trends (Fig. 2C).

**Table 1:** Summary of the characteristic DC-potential parameters analyzed for the various experimental conditions.

Treatment	Amplitude (ΔV)	Onset (Δt)	Duration (t ½)	n
ACSF, all 1^st^ HSDs	−15.8±3.9 mV	162.6±52.9 s	59.4±23.3 s	52
ACSF, 1^st^ HSD	−15.6±3.6 mV	147.2±50.8 s	65.7±9.9 s	7
ACSF, 2^nd^ HSD	0.93±0.11	1.03±0.10	0.95±0.19	7
ACSF, 3^rd^ HSD	0.83±0.23	1.09±0.20	0.88±0.13 *	6
Hypotonic	1.23±0.36	0.92±0.19	1.22±0.18 *	7
Hypertonic	0.72±0.25 *	1.25±0.18 *	1.07±0.60	6
Ca^2+^-free	0.85±0.28	0.75±0.18 **	0.58±0.44 *	6
Low Cl^−^	0.78±0.19 *	0.86±0.38	1.05±0.19	7
CN^−^ (10 µM)	0.96±0.15	0.82±0.15 **	1.19±0.22 *	8
CN^−^ (1 mM)	0.87±0.10 **	1.89±0.69 *	1.37±0.79	5
FCCP (10 µM)	0.89±0.27	0.30±0.10 **	0.57±0.32 **	6

The averaged absolute DC potential parameters of all control HSDs recorded are summarized in the first line. The second line shows the absolute DC potential parameters of those slices undergoing repeated hypoxia. All corresponding parameters determined upon drug treatment or repeated hypoxia were normalized to the respective control HSD recorded previously in each slice. Only these normalized parameters are shown to highlight the effects of a particular treatment. Asterisks indicate significantly different changes of the normalized data as compared by one-sample t-tests to unity (* P<0.05, ** P<0.01).

### Modulation of the IOS by Changes in Osmolarity

Since the occurrence of HSD and its optical signature are markedly affected by changes in osmolarity [20], [45], [46], we also analyzed the effects of hypotonic and hypertonic solutions on the multispectral IOS.

**Figure 3:**
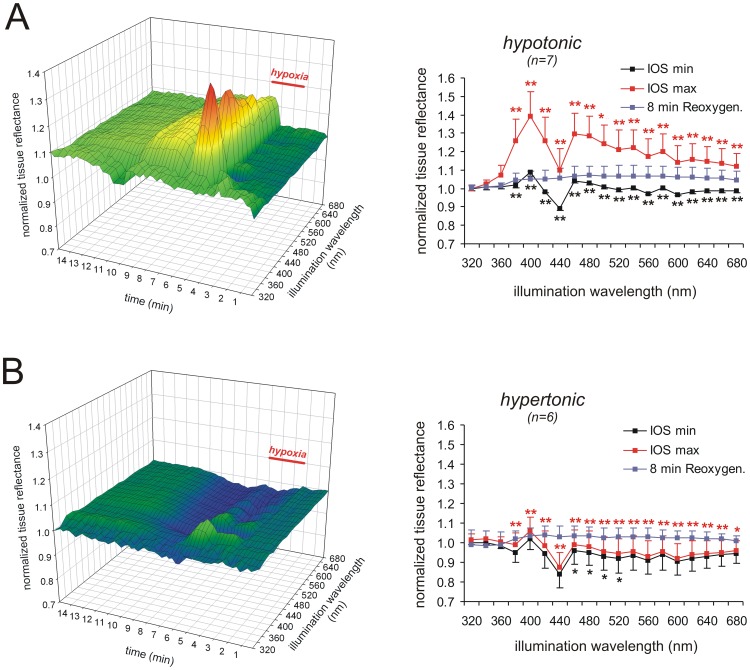
Hypotonicity intensifies whereas hypertonicity attenuates the multispectral IOS. A ) Inducing HSD in hypotonic solutions (30 mM NaCl omitted) intensified the multispectral IOS. As indicated by the averaged reflectance profiles, tissue reflectance within almost the entire wavelength range became markedly more intense during HSD. Color-matched asterisks indicate statistically significant changes as compared to the earlier recorded control HSDs (* P<0.05, ** P<0.01). **B**) In hypertonic solutions (60 mM mannitol added), the multispectral IOS was severely affected. Especially the reflectance increase during HSD was almost abolished.

In the presence of hypotonic solutions (30 mM NaCl omitted from the ACSF) the DC potential shift tended to occur earlier and its duration was slightly increased (Table 1, n = 7). The initial darkening of the slice shortly before HSD onset was less intense. Yet the IOS during HSD became almost doubled in intensity (Fig. 3A), and tissue reflectance at the two pronounced peaks of 400 and 460 nm now increased by 39.1±13.5% and 29.5±11.3% (n = 7), respectively. The nadir at 440 nm was still present but even here tissue reflectance now slightly increased by 10.1±11.3% above its normoxic baseline. Upon reoxygenation, recovery of the reflectance increase was slower and less complete than in control solutions, directly leading to the secondary increase in tissue reflectance during posthypoxic recovery.

Hypertonic solutions (60 mM mannitol added to the ACSF) postponed the onset of the DC potential shift, decreased its amplitude (Table 1, n = 6), and completely distorted the multispectral IOS (Fig. 3B, n = 6). The darkening of the tissue before HSD onset became slightly more pronounced. More importantly, the DC potential shift during HSD was no longer accompanied by the characteristic reflectance increase. Only with short wavelength illumination (<400 nm) a weak increase in tissue reflectance still occurred, whereas in the remaining spectrum reflectance decreased. The nadir at 440 nm was intensified, and tissue reflectance now decreased by −12.4±7.4% (n = 6). Upon reoxygenation, the reflectance decrease recovered to the prehypoxic baseline and it was then followed by a moderate secondary reflectance increase that did not differ from control conditions (Fig. 3B).

### The IOS Resists Glial Poisoning

The massive cellular depolarizations underlying HSD are generated by neurons, whereas glial cells follow passively due to massive uptake of K^+^ [3], [47]. Nevertheless, we pretreated slices with fluoroacetate (FAc, 5 mM, at least 3 h) to screen for a particular contribution of glial cells to the multispectral IOS. FAc is taken up by glial cells and converted into fluorocitrate which then blocks the citric acid cycle [48], [49]. Hence its inhibitory effects are - at least for the first couple of hours - largely restricted to glial cells [50], [51].

**Figure 4:**
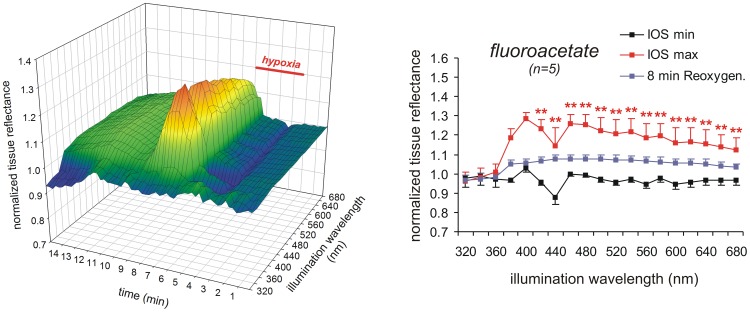
Glial poisoning does not oppose the generation of the IOS. Pretreatment of slices with FAc (5 mM) markedly intensified the reflectance increase during HSD. The nadir at 440 nm was dampened and the posthypoxic recovery slowed down.

Upon FAc pretreatment, the DC potential shift occurred within 2.7±0.5 min of O_2_ withdrawal, its amplitude averaged −18.6±3.6 mV and it lasted 17.9±18.8s (n = 5). As compared to the control HSDs (see Table 1) the duration of the DC potential shift was shortened whereas its time to onset and amplitude remained unchanged. The initial darkening of the tissue before HSD onset tended to be dampened. The reflectance increase during HSD was markedly intensified at wavelengths ≥420 nm (Fig. 4). At the two peaks of 400 and 460 nm tissue reflectance increased by 28.5±3.2% and 25.9±4.8%, respectively, and even at the 440 nm nadir it now increased by 14.7±9.1% (n = 5). The posthypoxic recovery of the IOS was incomplete and the secondary increase in reflectance was longer lasting than in control solutions (Fig. 4).

### Modulation of the IOS by Ion Substitution

In the next set of experiments we screened for the contribution of certain ion fluxes to the generation of the different components of the IOS and pretreated slices for 15–20 min with Ca^2+^-free solutions or low Cl^−^ solutions.

**Figure 5:**
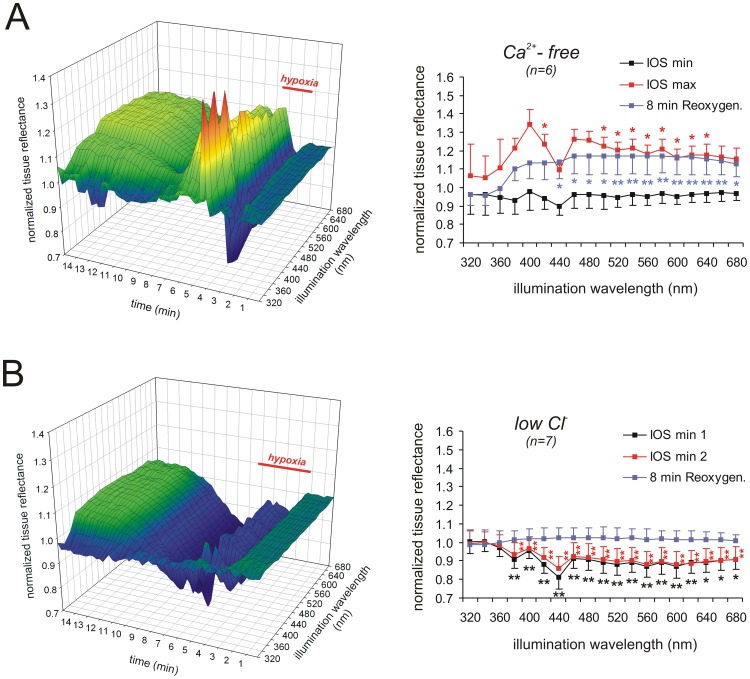
Ca^2+^-withdrawal intensifies, whereas Cl^–^replacement reverses the IOS. A ) Withdrawal of extracellular Ca^2+^ intensified the reflectance increase over large parts of the wavelength range during HSD and the posthypoxic recovery. Note that upon reoxygenation tissue reflectance recovered and was then followed by an irreversible secondary reflectance increase, suggesting dendritic damage. **B**) Near-complete replacement of Cl^−^ distorted the multispectral IOS. Instead of a reflectance increase HSD was now accompanied by a pronounced reflectance decrease. Nevertheless, a secondary reflectance increase occurred during posthypoxic recovery.

Withdrawal of extracellular Ca^2+^ hastened the onset of the DC potential shift and decreased its duration (Table 1; n = 6). The darkening of the tissue before HSD onset tended to be more pronounced, and the reflectance increase during HSD was markedly intensified (Fig. 5A). At the peaks of 400 and 460 nm reflectance now increased by 34.4±7.9% and 26.3±5.3%, respectively; even at the 440 nm nadir it increased slightly by 9.5±2.8% (n = 6). Upon reoxygenation, the IOS recovered and was then followed by the secondary reflectance increase. This secondary increase was more intense than under control conditions, showing a 16–17% reflectance increase at wavelengths of 460–640 nm even 8 min after reoxygenation, and it did not noticeably recover during the remaining course of the experiment (Fig. 5A).

Treatment with low Cl^−^ solutions (∼95% of Cl^−^ were replaced by methylsulfate) decreased the amplitude of the DC potential shift (Table 1; n = 7) and the IOS was markedly modified (Fig. 5B). The initial darkening before HSD onset was clearly intensified, and the nadir at 440 nm showed a reflectance decrease by 18.9±6.5% (n = 6). During HSD an increase in tissue reflectance did not occur. Instead, darkening of the tissue continued (plotted as IOS min 2 in Fig. 5B), reaching a total reflectance decrease by 13.8±6.0% (n = 6) at 440 nm. Upon reoxygenation, the reflectance decrease recovered to the normoxic baseline and it was then followed by the moderate secondary reflectance increase (Fig. 5B).

### Modulation of the IOS by Mitochondrial Inhibition

Earlier studies suggested a contribution of mitochondria to the generation of the IOS associated with SD [16], [26], [34]. Furthermore, the changes in tissue reflectance at short wavelengths (380–440 nm) are the most intense components of the IOS and in part start early during hypoxia even before HSD onset. We therefore asked, whether these components may arise from changes in mitochondrial activity/metabolism and induced HSD in the presence of mitochondria-directed drugs.

**Figure 6:**
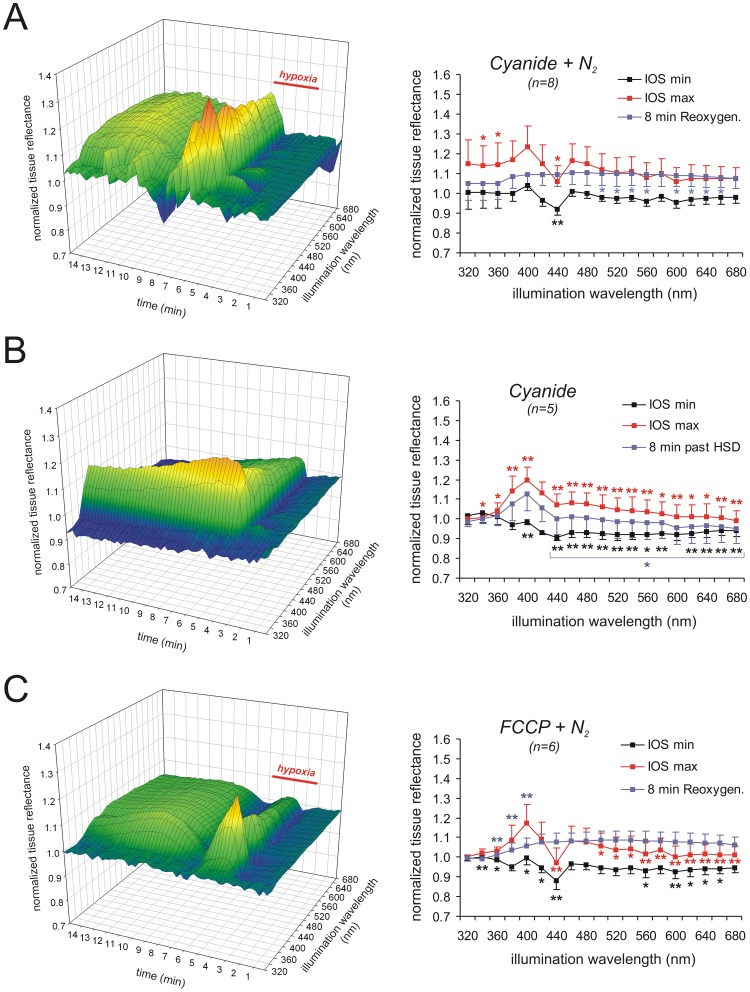
Mitochondrial metabolism dominates the short-wavelength components of the IOS. A ) Inducing HSD in the presence of 10 µM CN^−^ did not cause marked alterations in the multispectral IOS or the averaged reflectance profiles. Only the 440 nm nadir was less pronounced and the posthypoxic secondary reflectance increase was prolonged. **B**) When HSD was induced directly by 1 mM CN^−^ (in the presence of O_2_) especially the reflectance increase at short wavelengths was intensified and the nadir at 440 nm was abolished; reflectance at longer wavelengths increased less intensely. Note that a recovery of the reflectance increase at short-wavelengths did not occur, and that a posthypoxic secondary reflectance increase at longer wavelengths was absent. **C**) Inducing HSD in the presence of FCCP (10 µM) caused a less intense reflectance increase especially at intermediate and longer wavelengths. Also the secondary reflectance increase during the posthypoxic recovery tended to become more intense and prolonged.

Treating slices with a low dose of CN^−^ (10 µM, 15 min) hastened the onset of HSD and increased the duration of the DC potential shift (Table 1; n = 8). Darkening of the tissue shortly before HSD onset was largely preserved but less intense at the nadir of 440 nm (−8.1±2.8%, n = 8). After the onset of HSD a clear increase in light reflectance occurred, which averaged 23.3±10.5% and 16.6±8.5% at 400 and 460 nm, respectively; the nadir at 440 nm was again less pronounced (Fig. 6A). Upon reoxygenation in the continued presence of 10 µM CN^−^, tissue reflectance recovered. The secondary reflectance increase tended to be more pronounced and longer lasting than under control conditions.

As shown earlier, HSD can be induced directly by high doses of CN^−^ despite the presence of O_2_ [26], [52]. CN^−^ blocks mitochondrial respiration at complex IV thereby preventing the utilization of O_2_, but it acts slower than O_2_ withdrawal. Accordingly, upon application of 1 mM CN^−^, the DC potential shift occurred markedly delayed, showed a decreased amplitude, and its duration tended to be increased as CN^−^ was not withdrawn after HSD had occurred (Table 1; n = 5). Such chemically-induced hypoxia was accompanied by a clearly different IOS profile. Early during CN^−^ administration before HSD onset, the reflectance peak at 400 nm and the nadir at 440 nm were dampened. Even though O_2_ was not withdrawn, tissue reflectance decreased at wavelengths ≥380 nm. After the onset of HSD, a marked increase in reflectance developed especially at the short wavelengths, averaging 19.7±6.6% at 400 nm (n = 5). Interestingly, the nadir at 440 nm was absent and at wavelengths >440 nm reflectance increased only slightly and less intensely as compared to control conditions (Fig. 6B). These reflectance changes were only partly reversible in the continued presence of CN^−^, and especially tissue reflectance at shorter wavelengths (380–420 nm) remained increased until the end of the experiment. A secondary reflectance increase at wavelengths ≥440 nm did not occur.

To screen for a mechanistic contribution of the mitochondrial membrane potential (ΔΨ_m_), we pretreated slices with the uncoupling agent FCCP (10 µM, 15 min), to rapidly dissipate ΔΨ_m_ as well as cellular ATP reserves (for review see: [53]). Mitochondrial uncoupling prior to O_2_ withdrawal markedly hastened the onset of the DC potential shift and shortened its duration (Table 1; n = 6). During the early phase of hypoxia light reflectance decreased, but as soon as HSD occurred, the clear increase in tissue reflectance was largely restricted to shorter wavelengths (Fig. 6C). At 400 nm the reflectance increase averaged 17.3±9.3%, and at 460 nm it tended to be less intense, averaging only 8.4±7.3% (n = 7). The recovery of the IOS upon reoxygenation in the continued presence of FCCP was comparable to control conditions, but the secondary reflectance increase tended to be more intense and longer lasting (Fig. 6C).

## Discussion

Even though the detailed mechanisms underlying the generation of the IOS are far from being understood completely, these signals serve as elegant and non-invasive optical tools to define the spatiotemporal propagation pattern of SD within brain tissue. However, it seems that in the past a lack of comparability of data sets obtained from various preparations under different conditions, may have contributed in part to the reporting of seemingly contradictory results. Hence, there is a clear need for an improved standardization of IOS recordings. A promising approach in terms of such standardization and more reliable and stable IOS imaging is the use of one or more clearly defined wavelengths.

### Multi-spectral Analyses of the IOS

There have already been efforts to analyze IOS components at various wavelengths, e.g., by performing the *in vivo* imaging of normoxic SD at 3 different wavelengths to separate the pronounced blood perfusion induced and hemoglobin-oxygenation related optical changes from the less intense alterations in light scattering [32]. Also in blood-free rat brain 3 different wavelengths were used to screen for the spectral composition of the scattering and absorption changes in global ischemia [33]. Others have applied different wavelengths to combine the monitoring of IOS with the imaging of additional cellular parameters such as intracellular Ca^2+^ changes [19] or ΔΨ_m_ [34]. Furthermore, there have been attempts to analyze spectrally the near-infrared light scattered back from rat brain to separate changes in the redox status of cytochrome c oxidase and oxygenated/deoxygenated hemoglobin [54]. In a similar approach optical imaging spectroscopy was used to obtain spectral information of the white light reflected from different cortical locations to define the interaction of neuronal activity and microcirculation upon sensory stimulation [55].

Here we present the first detailed multispectral analyses of the IOS accompanying hypoxia and HSD. The rapidly switchable light source used, provides the entire visible spectrum and even beyond (320–680 nm). Based on its spectral bandwidth (15 nm at half-power), the exposure time required for a single image (30 ms were chosen to prevent too intense pixel noise of the CCD chip), and the time allocated for wavelength switching (100 ms), we decided to change illumination wavelengths in 20 nm increments and ended up with 19 different wavelengths for the multispectral imaging of HSD in hippocampal slices.

### Mechanism of the IOS

The major components of the IOS arise from: 1) metabolically-related changes in the absorption/fluorescence of intrinsic chromophores such as the cytochromes, 2) activity-induced alterations in blood volume, 3) activity-related changes in hemoglobin oxygenation, and 4) modified light scattering on the cellular and organelle level [15], [17], [33], [56], [57], [58]. In our experiments we used acute hippocampal slices, i.e., an isolated blood free preparation. Therefore, any influence of blood flow changes can be ruled out. In isolated tissue, the main light absorbing chromophores besides hemoglobin are the cytochromes and cytochrome c oxidase [33]. Accordingly, in rat brain after blood removal the IOS associated with global ischemia was reported to consist of a metabolic component and to reflect changes in cellular organelles, especially increases in the size of mitochondria, as well as dendritic changes [33].

One might argue that the oxygenation status of the hemoglobin remaining in isolated tissue could be affected by changes in neuronal activity or O_2_ supply. Indeed earlier reports showed that residual hemoglobin decreased the absolute intensity of NADH autofluorescence by partly absorbing the 366 nm excitation light, but it did not modify the general shape of the emission spectrum [58]. Further convincing evidence against a prominent contribution of the oxygenation level of hemoglobin arises from the observation that CN^–^induced HSD evoked the characteristic increase in tissue reflectance despite the presence of O_2_. The marked effects of the osmolarity changes and especially the “reversal” of the IOS by hypertonic and low Cl^−^ solutions, which are in accordance to earlier studies [16], [44], [59], also argue against a marked contribution of changes in hemoglobin oxygenation or oxidation/reduction of cytochromes. They rather point out to a major influence of ion and water movements, i.e., changes in light scattering, in the generation of the IOS.

Tissue autofluorescence is dominated by NADH, whereas FAD contributes only to a minor degree [58]. Since NADH levels increase during hypoxia and HSD [60], one may assume that also NADH autofluorescence contributes to the IOS. Yet, autofluorescence is far too weak to contribute noticeably. Its monitoring requires intense excitation and an efficient separation of excitation and emission, none of which was performed here. Another argument against a contribution of NADH autofluorescence is that even in the presence of FCCP, which decreases NADH fluorescence [26] by stimulating mitochondrial respiration, a pronounced reflectance increase occurred at ∼400 nm, i.e., the emission wavelength of NADH. Yet, absorption changes could contribute by modulating light reflectance in the tissue under investigation.

### Components of the Multispectral IOS

In accordance to observations in the isolated retina during normoxic SD [44], the four clearly distinguishable phases of the IOS were also present in the multispectral IOS. Phase 1, the reflectance decrease before HSD onset, is considered to arise from cell swelling [16], [20], and it was present within almost the entire wavelength range. Since this initial darkening tended to be less intense upon FAc poisoning, one may assume that glia contributes to the early cell swelling. An obvious cause might be the glial uptake of K^+^ released by neurons. It is evident as an early glial depolarization already before HSD onset [61] and hence could lead to the early cell swelling observed during interstitial volume measurements with TMA^+^-selective microelectrodes [16], [41].

Phase 2, the reflectance increase accompanying the DC potential deflection, is the most pronounced but also most complex phase. It is clearly generated by more than a single underlying mechanism. In line with earlier studies, Ca^2+^-withdrawal did not prevent the occurrence of SD [19], [40] or the accompanying IOS [34], but rather intensified this phase of the IOS. Accordingly, the massive Ca^2+^ influx during SD and its downstream effects cannot be a cause of the pronounced reflectance increase. The mid-wavelength components (>460 nm) of the reflectance increase strictly depend on Cl^−^ and at least in part on an intact ΔΨ_m_, which verifies that mitochondria contribute to these optical changes.

A marked characteristic of phase 2 is the nadir at 440 nm. It was clearly visible under all paradigms, even in low Cl^−^ solutions which reversed the reflectance increase. This strongly suggests that the 440 nm nadir represents a massive absorption band referred to as Soret band, i.e., the γ absorption peak of porphyrins. The pronounced light absorption by the cytochromes [35], [36] and oxy/deoxyhemoglobin at this wavelength [62] is that intense that it dominates the reflectance changes during HSD at 440 nm.

Another characteristic of phase 2 is the pronounced reflectance increase at 400 nm, which is the absolute intensity peak of the IOS. It is still partly present in hypotonic solutions but fully blocked in low Cl^−^ solutions, indicating that also here ion and water movements are crucial. Yet, in contrast to the other spectral components, the 400 nm peak becomes irreversible when HSD is induced by CN^−^, i.e., during maintained inhibition of mitochondrial respiration, but not upon FCCP treatment which dissipates ΔΨ_m_ and thereby stimulates respiration. Accordingly, the 400 nm peak arises from alterations in mitochondrial metabolism or function triggered by massive ion fluxes and ATP shortage during hypoxia and HSD. A mere depolarization of mitochondria - as induced by uncoupling - is not a sufficient cause.

Phase 3, the recovery of the prior reflectance changes upon reoxygenation, was hardly affected by the various treatments, except when HSD was induced by CN^−^ which then remained in the tissue. Therefore, this phase which was not analyzed in more detail represents the normalization of cellular (neuronal and glial) membrane potentials and ion homeostasis by the reinstatement of mitochondrial respiration.

Phase 4, the secondary reflectance increase occurring several minutes upon reoxygenation, was present at wavelengths ≥380 nm without showing any further spectral characteristics. Based on our earlier cellular and ionic analyses performed under very similar experimental conditions, neuronal and glial membrane potentials and ionic distribution can be expected to be mostly reinstated at that time [61]. This phase was intensified and less reversible only after very intense and highly synchronized HSD events, e.g., upon Ca^2+^-withdrawal or glial poisoning. Hence, it is independent of Ca^2+^ influx and mostly of neuronal origin. Also, it is the only phase of the IOS that was not affected by low Cl^–^solutions.

Earlier light microscope studies revealed that the diameter of apical dendrites increases during cortical SD [63], which apparently is a consequence of their marked depolarization. Such structural alterations (beading of dendrites) were proposed to contribute to the optical signatures of HSD and ischemic depolarizations [17]. This concept would fit to the increased ultrastructural damage that might be expected upon intensified HSD episodes. Yet, on the other hand, phase 4– especially its mid-wavelengths components (λ>460 nm) – tended to be dampened by high doses of CN^−^, i.e., lack of cellular recovery. This may support the concept that metabolic alterations, required to complete the recovery of membrane potentials and ionic distribution after HSD, could as well contribute to this late phase of the IOS [44].

At very short illumination wavelengths (320–340 nm) hardly any changes were observed during the four IOS phases. Though being delivered via a quartz fiber, the scattered light of these very short wavelengths can be assumed to be largely absorbed by the optics. According to manufacturer information, the 5X objective used lacks a noticeable transmission at wavelengths <350 nm; typical transmission at 360 and 380 nm is ∼15% and ∼70%, respectively, and in the range of 400–680 nm it is >90%. Therefore, the contribution of wavelengths <360 nm to the spectral signature of the IOS cannot be rated.

### Contribution of Mitochondria

Earlier we suggested that it may be alterations in mitochondrial structure that contribute to the reflectance increase accompanying the DC potential shift [16]. Since mitochondrial swelling is induced by Ca^2+^ [64], we have now tested for the effects of Ca^2+^-free solutions, but found an intensified IOS during HSD and posthypoxic recovery. Another interesting fact is that even in isolated brain mitochondria, anoxia causes matrix shrinkage and a reversible increase in light scattering [65]. These changes are due to alterations in matrix space, not total mitochondrial volume, and their magnitude is ATP independent, but ATP slows down its time course. Since inhibitors of electron transport also increased mitochondrial light scattering [65], it was suggested that these optical changes are mainly caused by the arrest of electron flow.

An earlier study [34] unveiled a close relation of ΔΨ_m_ (recorded as Rh123 fluorescence) and the IOS. Hypoxia already before HSD onset caused a gradual mitochondrial depolarization, and HSD elicited a sharp increase in Rh123 fluorescence. This sharp Rh123 increase was attenuated by low Cl^−^ solutions but not Ca^2+^-withdrawal [34]. During posthypoxic recovery a moderate secondary increase in Rh123 fluorescence occurred, that was intensified upon prolonged hypoxia. Normoxic SD elicited much less intense Rh123 increases and no early changes occurred [34]. It therefore appears that mitochondria respond to both, O_2_ withdrawal and the metabolic load associated with massive neuronal/glial depolarizations during SD, and that they contribute during both of these conditions to the various phases of the IOS.

### Concluding Remarks

The multispectral analyzes performed yielded more detailed insights into those mechanisms generating the IOS during the course of HSD. Despite its multiphasic nature and complex spectral composition the IOS is strictly Cl^–^dependent, but does not rely on Ca^2+^ influx. In terms of reliability and stability, the mid-range of the visible spectrum is recommended, i.e., wavelengths of 460–560 nm. These reflectance changes are somewhat less intense than those at shorter wavelengths, but they are dominated by light scattering and less affected by alterations in mitochondrial function. The short wavelength components of the IOS (<420 nm) are dominated by mitochondrial metabolism and function. The massive absorption band at 440 nm represents the Soret peak, the absorption maximum of porphyrins, which masks the reflectance increases associated with HSD. Therefore, especially this wavelength range should be avoided for the optical monitoring of HSD propagation in brain tissue.
